# Biallelic loss of *LDB3* leads to a lethal pediatric dilated cardiomyopathy

**DOI:** 10.1038/s41431-022-01204-9

**Published:** 2022-10-17

**Authors:** Tamara T. Koopmann, Yalda Jamshidi, Mohammad Naghibi-Sistani, Heleen M. van der Klift, Hassan Birjandi, Zuhair Al-Hassnan, Abdullah Alwadai, Giovanni Zifarelli, Ehsan G. Karimiani, Sahar Sedighzadeh, Amir Bahreini, Nayereh Nouri, Merlene Peter, Kyoko Watanabe, Hermine A. van Duyvenvoorde, Claudia A. L. Ruivenkamp, Aalbertine K. K. Teunissen, Arend D. J. Ten Harkel, Sjoerd G. van Duinen, Monique C. Haak, Carlos E. Prada, Gijs W. E. Santen, Reza Maroofian

**Affiliations:** 1 Department of Clinical Genetics/LDGA, Leiden University Medical Center, Leiden, The Netherlands; 2 Genetics Research Centre, Molecular and Clinical Sciences Institute, St George’s University of London, London, UK; 3 Pediatric & Congenital Cardiology Division, Pediatric Department, Faculty of Medicine, Mashhad University of Medical Sciences, Mashhad, Iran; 4 The Cardiovascular Genetics Program, Centre for Genomic Medicine, King Faisal Specialist Hospital & Research Center, Riyadh, Saudi Arabia; 5 PICU Department, Prince Sultan Cardiac Center, Riyadh, Saudi Arabia; 6 CENTOGENE GmbH, Am Strande 7, 18055 Rostock, Germany; 7 Department of Medical Genetics, Next Generation Genetic Polyclinic, Mashhad, Iran; 8 Department of Biological Sciences, Faculty of Science, Shahid Chamran University of Ahvaz, Ahvaz, Iran; 9 KaryoGen, Isfahan, Iran; 10 Department of Human Genetics, Graduate School of Public Health, University of Pittsburgh, Pittsburgh, PA USA; 11 Department of Genetics and Molecular Biology, School of Medicine, Isfahan University of Medical Sciences, Isfahan, Iran; 12 Division of Genetics, Birth Defects & Metabolism, Ann & Robert H. Lurie Children’s Hospital of Chicago, Chicago, IL 60611 USA; 13 Division of Cardiology, Ann & Robert H. Lurie Children’s Hospital of Chicago, Chicago, IL 60611 USA; 14 Department of Obstetrics and Prenatal Diagnosis, Leiden University Medical Center, Leiden, The Netherlands; 15 Department of Pediatric Cardiology, Willem Alexander Children’s Hospital, Leiden University Medical Center, Leiden, The Netherlands; 16 Department of Pathology, Leiden University Medical Center, Leiden, The Netherlands; 17 Department of Pediatrics, Feinberg School of Medicine of Northwestern University, Chicago, IL 60611 USA; 18 Department of Neuromuscular Disorders, Queen Square Institute of Neurology, University College London, London, UK

**Keywords:** Genetics research, Next-generation sequencing, Cardiomyopathies

## Abstract

Autosomal dominant variants in *LDB3* (also known as ZASP), encoding the PDZ-LIM domain-binding factor, have been linked to a late onset phenotype of cardiomyopathy and myofibrillar myopathy in humans. However, despite knockout mice displaying a much more severe phenotype with premature death, bi-allelic variants in *LDB3* have not yet been reported. Here we identify biallelic loss-of-function variants in five unrelated cardiomyopathy families by next-generation sequencing. In the first family, we identified compound heterozygous LOF variants in *LDB3* in a fetus with bilateral talipes and mild left cardiac ventricular enlargement. Ultra-structural examination revealed highly irregular Z-disc formation, and RNA analysis demonstrated little/no expression of LDB3 protein with a functional C-terminal LIM domain in muscle tissue from the affected fetus. In a second family, a homozygous *LDB3* nonsense variant was identified in a young girl with severe early-onset dilated cardiomyopathy with left ventricular non-compaction; the same homozygous nonsense variant was identified in a third unrelated female infant with dilated cardiomyopathy. We further identified homozygous *LDB3* frameshift variants in two unrelated probands diagnosed with cardiomegaly and severely reduced left ventricular ejection fraction. Our findings demonstrate that recessive *LDB3* variants can lead to an early-onset severe human phenotype of cardiomyopathy and myopathy, reminiscent of the knockout mouse phenotype, and supporting a loss of function mechanism.

## Introduction

Genetically inherited cardiomyopathies are a highly heterogeneous group of disorders characterized by structural and/or functional disturbances in the myocardium [1]. Variants in genes encoding sarcomeric proteins, the contractile units of striated muscle cells, are a leading cause of cardiomyopathies [2].

Z-discs delineate the lateral borders of sarcomeres and are the smallest functional units in striated muscle. Z-disc proteins have been implicated in both inherited skeletal and cardiac muscle diseases or can cause myopathy with cardiac involvement [3].

The conserved proteins α-actinin and Z-disc Alternatively Spliced Protein (ZASP) are present at the earliest stages of Z-disc formation and are required for Z-disc assembly [4, 5]. ZASP is required to stabilize the sarcomere during contraction, through interactions with actin in cardiac and skeletal muscles [5].

ZASP is encoded by PDZ-LIM domain-binding factor encoding gene *LDB3*, also known as *Cypher* and *Oracle*. *LDB3* contains a PDZ domain, located at the N-terminus, and an internal ZASP/cypher-like motif (ZM) both capable of interacting with α-Actinin-2 [5]. Additionally, the PDZ domain interacts with Myotilin and FATZ, which provides structural stability to the Z-disc [6]. The C-terminus contains three LIM domains, which bind to protein kinase C and are therefore speculated to play a role in protein kinase C-mediated signaling [7].

Heterozygous missense variants in *LDB3* (MIM: 605906) have been associated with a range of myopathies including cardiomyopathy [8] and myofibrillar myopathy (MFM) [9–11]. However, bi-allelic and truncating variants have not yet been reported. In mice however global and cardiac specific knockout models have an extremely early-onset myopathy/cardiomyopathy, and ultrastructural examination show disorganized and fragmented Z-lines [12, 13].

Here we report for the first time 5 families with homozygous or compound heterozygous loss-of-function variants and a severe phenotype compatible with that reported in mice.

## Methods

**Fig. 1:**
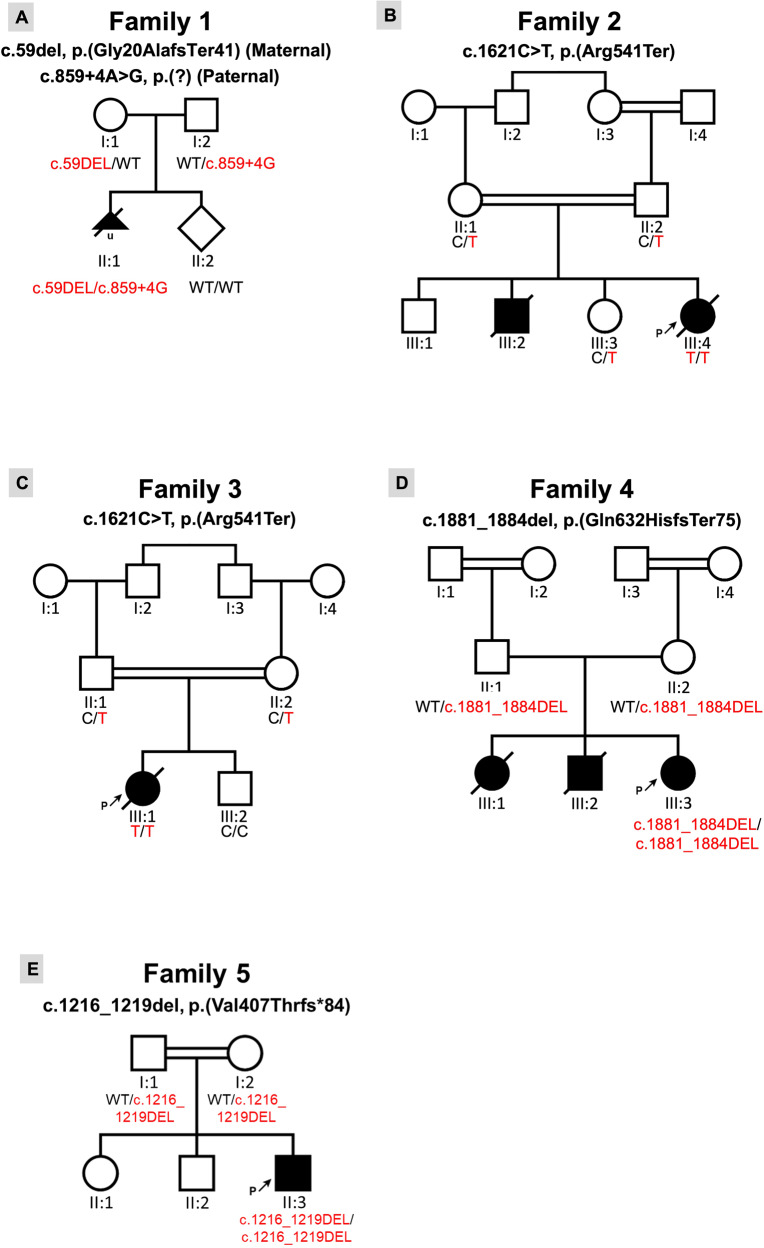
Pedigrees of families with bi-allelic LDB3 loss-of-function variants. **A** Pedigree of family 1. The proband, II:1 is compound heterozygous for an LDB3 deletion and an LDB3 splice variant. **B** Pedigree of family 2, a consanguineous family from Iran. The proband, III:4, is indicated with a black arrow, and is homozygous for an ultra-rare nonsense variant. **C** Pedigree of family 3, a consanguineous family from Saudi Arabia. The proband, III:1, is indicated with a black arrow, and is homozygous for the same nonsense variant as in family 2. **D** Pedigree of family 4, a consanguineous family from Iran. The proband, III:3, is indicated with a black arrow, and is homozygous for a novel LDB3 frameshift variant. **E** Pedigree of family 5, a consanguineous family from Mexico. The proband, II:3, is indicated with a black arrow, and is homozygous for a novel LDB3 frameshift variant.

We evaluated four consanguineous Middle Eastern families and one non-consanguineous West-European family identified through data sharing with colleagues and using GeneMatcher [14] (Fig. 1A–E). Detailed clinical features as well as family history were obtained from all affected individuals and reviewed. Clinical and research whole exome sequencing (WES), as well as Sanger sequencing, were performed independently. Briefly, variants were annotated for variant consequence, SIFT scores, PolyPhen scores, CADD scores, and allele frequencies in the 1000 Genomes populations, GnomAD allele frequencies, and relevant local databases (e.g., the Genome of the Netherlands frequencies (GoNL) for Family 1; the Greater Middle East (GME) Variome Project frequencies, and in-house ethnically matched databases). After annotation, variants with an allele frequency of >1% were excluded from further interpretation. For Family 1 MOON software (Diploid, Leuven, Belgium) was used for interpretation of variants with Human Phenotype Ontology (HPO) terms: Cardiomegaly HP:0001640; Bilateral talipes equinovarus HP:0001776 followed by analysis of variants in genes from a virtual cardiomyopathy panel: *ACTC1 ACTN2 ALPK3 ANKRD1 BAG3 CALR3 CAV3 CDH2 CRYAB CSRP3 CTNNA3 DES DSC2 DSG2 DSP EMD FHL1 FHL2 FKRP FLNC GLA HCN4 JPH2 JUP LAMA4 LAMP2 LDB3 LMNA MIB1 MYBPC3 MYH6 MYH7 MYL2 MYL3 MYLK3 MYOZ2 MYPN NEXN PKP2 PLN PPA2 PRDM16 PRKAG2 RBM20 SCN5A TAZ TCAP TMEM43 TNNC1 TNNI3 TNNT2 TPM1 TTN TTR VCL*. For Families 2-5 only homozygous variants falling within a region of homozygosity (using AutoMap (https://automap.iob.ch/)) in genes from the virtual cardiomyopathy panel were retained. The variant classification and interpretation were performed in accordance with the clinical standards of the American College of Medical Genetics and Genomics/Association of Molecular Pathology (ACMG/AMP) guidelines [15]. Since we are providing evidence that loss-of-function variants in the *LDB3* gene are associated with disease, we did not apply PVS1. A clinical SNP array for Family 1 was performed using the Affymetrix CytoScan HD Array according to manufacturer’s protocol (Thermo Fisher Scientific Inc., CA, USA).

### RNA study

For Family 1, total RNA was isolated from skeletal muscle biopsy of the affected fetus (tibialis anterior), a fetal negative control (fetus of comparable age, psoas muscle), and an adult negative control (skeletal muscle, not further specified) using the NucleoSpin RNA XS kit (Machery-Nagel), according to the manufacturer’s protocol. A total of 250 ng RNA was reverse transcribed in a total volume of 20 µl using random hexamer primers (Roche Diagnostics) and Superscript II RNase H Reverse Transcriptase (Invitrogen Life Technologies). PCR amplification was performed on 1 µl cDNA in a 15 µl total volume using GoTaq hotstart DNA polymerase (Promega); annealing temperature 60 °C, 35 cycles.

*LDB3* encodes several alternatively spliced isoforms including a short (missing the C-terminal LIM domain) and long isoform [16]. RNA-seq data available in the GTEx database (last accessed on 31 August 2020) indicated that three of the nine alternative isoforms show high expression in (adult) skeletal muscle tissue and our RT-primer design was based on the structure of these isoforms.

Amplicons specific for the long or the short LDB3 isoforms were designed (primer sequences available on request). Electrophoresis was performed using ethidium bromide-stained 1% agarose gels. PCR products were sequenced on an Applied Biosystems 3730xl DNA analyzer using M13 primers. Sequence results were analysed using CodonCode Aligner software and Chromas (Technelysium Pty Ltd).

## Results

### Family 1

A G1P0 32-year-old pregnant woman was referred because talipes were detected during a routine structural ultrasound. Extensive ultrasound examination at 19 weeks and 2 days gestation revealed bilateral talipes and cardiomegaly (Cardiothoracic Ratio CTR 0.62), with enlargement of mainly the left ventricle and atrium. The heart was structurally normal, without decompensation. Amniocentesis was performed for prenatal genetic testing. Chromosomal analysis of amniocytes performed by single nucleotide polymorphism array came back normal with was no evidence of abnormal copy number in the fetus (arr(1–22)x2,(X, Y)x1). Exome sequencing of fetal DNA from amniotic fluid indicated two novel heterozygous variants in the *LDB3* gene NC_000010.11(NM_007078.2):c.859 + 4 A > G and NM_007078.2:c.59del (Fig. 1A). Sanger sequencing revealed that the c.59del was inherited from the mother and leads to a premature termination codon NP_009009.1:p.(Gly20AlafsTer41). The paternally inherited intronic variant c.859 + 4 A > G; NP_009009.1:p.? was predicted to affect splicing using splice prediction tools integrated in software package Alamut Visual version 2.11 (Interactive Biosoftware, Rouen, France). Both variants are present in the long and short isoforms of LDB3. WES did not identify any additional variants of interest in Family 1. According to ACMG guidelines both variants were assigned Likely Pathogenic status (c.59del p.(Gly20Alafs*41): PM2; PM3; PS3: LP c.859 + 4 A > G: PM2; PM3; PS3: LP).

Although no human phenotype of *LDB3* knockout was known, we considered the phenotype in this fetus similar enough to the preclinical data to discuss the findings, albeit with great care. After extensive counseling, the parents decided to terminate the pregnancy at gestational age of 23 weeks and 2 days based on the potentially severe postnatal phenotype. Post-mortal examination was performed. Both parents declined cardiac examination.

**Fig. 2:**
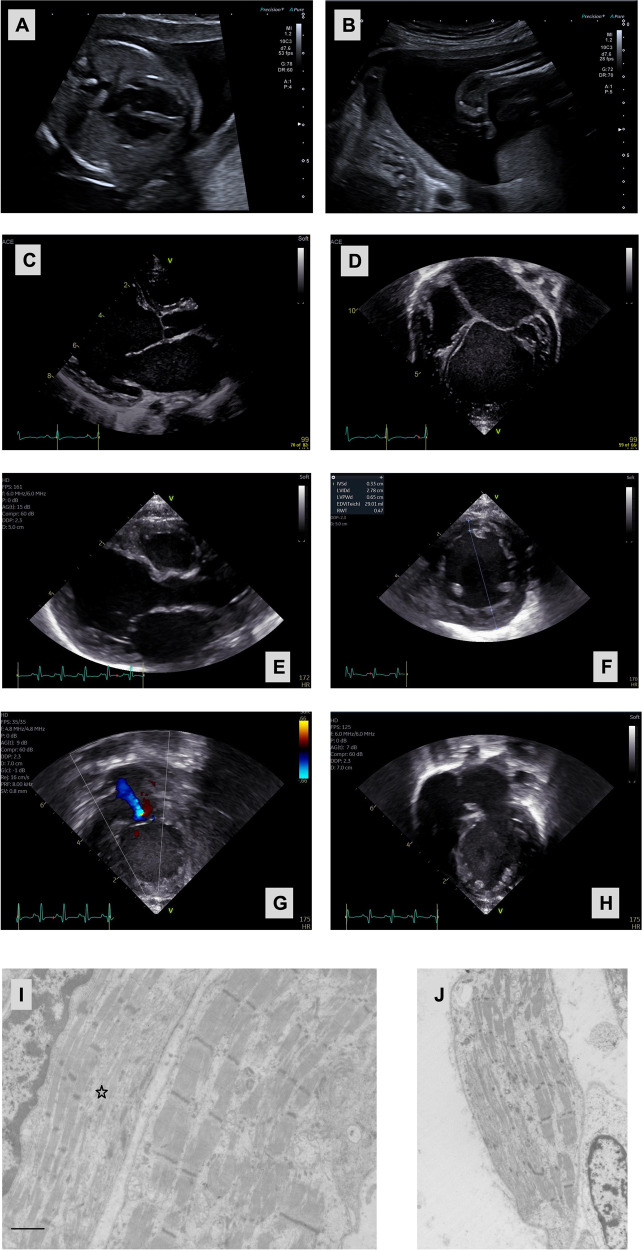
Cardiac imaging and electron microscopy from individuals with bi-allelic LDB3 loss-of-function variants. Advanced ultrasound imaging of the fetus (proband) from family 1 at gestational age of 21 weeks: four chamber view revealing **A** moderate dilatation of the left ventricle; **B** lower leg in frontal view showing clubfoot. **C** Echocardiography of proband from family 3 revealing a very dilated left ventricle and left atrium in the parasternal long-axis view, close up revealing very dilated left atrium (**D**). Echocardiography from proband of family 5: **E** Parasternal long access view of moderate left ventricular dilation, **F** Parasternal short access view of moderate left ventricular dilation, **G** Apical 2 chamber view of moderate mitral valve regurgitation, **H** Apical 4 chamber view of moderate left ventricular dilation with notable apical trabeculations. **I**, **J** Electron microscopy of the muscle fibers of both the quadriceps femoris and the tibialis anterior muscle from the fetus from family 1 show variable disorganisation of the myofibrils, especially of the Z lines, with partial loss of myofibrils. The bar is 1 µm; the star indicates a fiber with clear disorganization of the Z lines.

Fetal autopsy of the fetus revealed multiple anomalies. Mild wrist contractures in addition to talipes were present; examination of the heart showed a hypertrophic left ventricle (Fig. 2A–D). As *LDB3* is also associated with MFM electron microscopy was performed for several muscle groups (tibialis anterior and quadriceps femoris) and showed highly irregular Z-disc formation and irregularity and possibly reduction in myofibrils (Fig. 2I, J). Microscopic examination of cardiac muscle was consistent with these results, but inconclusive since muscle degradation had already started.

**Fig. 3:**
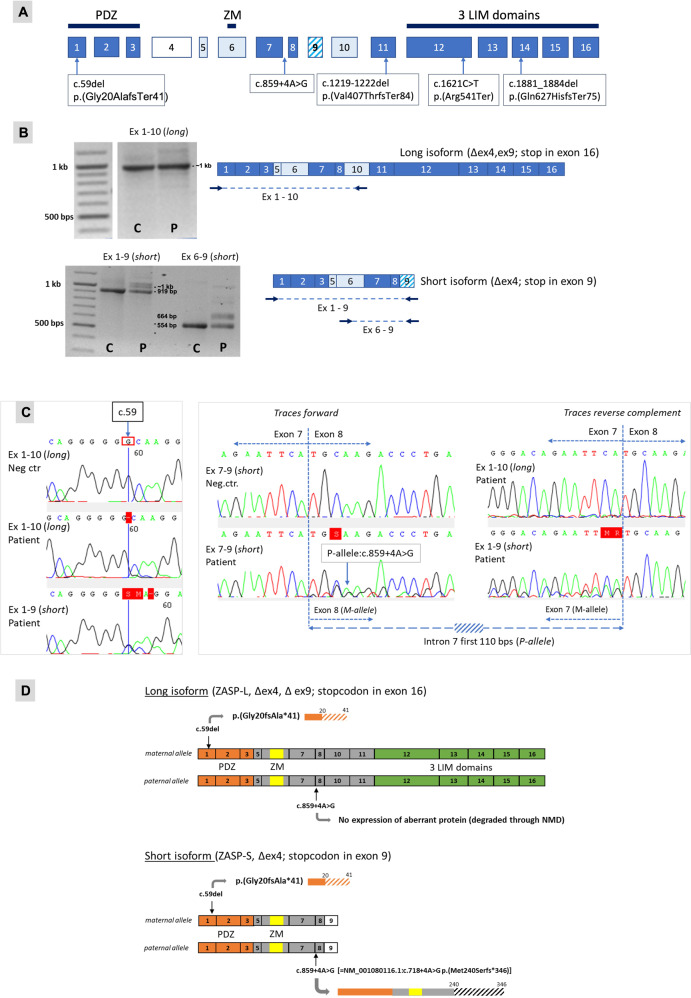
RNA analysis of genomic LDB3 variants and predicted protein expression performed on RNA isolated from fetal muscle (family 1). Variant in paternal allele: LRG_385:g.28506 A > G, long isoform NM_007078.2:c.859 + 4 A > G, short isoform NM_001080116.1:c.718 + 4 A > G, GRCh37 location: 88451826. Variant in maternal allele: LRG_385:g.5187del, NM_007078.2:c.59del, GRCh37 location: 88428507. Primer sequences available on request. **A** Schematic presentation of the LDB3 gene; exons 4, 5, 6, 9, and 10 are alternatively spliced (exon 4 mainly expressed in heart, exon 9 only in short isoforms, exon 10 present in prenatal and absent in postnatal skeletal muscle). Location of the pathogenic variants (nomenclature with reference to NM_007078.2) found in family 1–5 is indicated. **B** RT-PCR results with primers that specifically amplify cDNA from either the long isoforms (fragment exons 1-10) or the short isoforms (fragments exons 1-6 and exons 6-9). Primer locations are indicated by arrows; C = fetal negative control, *P* = patient fetus. Fragment exons 1-10 does not show any aberrant band on agarose gel; presumably because the aberrant paternal transcript is degraded by Nonsense-mediated RNA decay (NMD). **C** Sequence chromatograms of RT-PCR products showing traces at maternal variant c.59del (left panel) and traces with aberrant splicing caused by the paternal intronic variant NM_007078.2:c.859 + 4 A > G (=NM_001080116.1:c.718 + 4 A > G in the short isoform; right panel) in family 1. (Left) RT-PCR with exon 1-10 primers (long isoform) show expression of patients’ maternal allele only; RT-PCR with exon 1-9 primers (short isoform) show bi-allelic expression at position c.59. (Right) In the sequence traces obtained from patients RT-PCR products with short isoform-specific primers an insertion of a 110 bp long intronic sequence is observed running along with the traces from the wildtype transcript. **D** Prediction of aberrant allele-specific protein expression based on the RNA analysis of the paternal and maternal LDB3 variants present in the DNA of the family 1 fetus.

RNA analysis was performed to evaluate the consequences on RNA splicing and expression of the two *LDB3* variants identified. RNA from the affected fetus and from a fetal as well as adult negative muscle control were used. It should be noted that the postnatal long LDB3 isoform differs from the prenatal form in lacking exon 10 (LRG-specific exon numbering; see Fig. 3A). RT-PCR of the proband showed expression of the long LDB3 transcript (ZASP-L) encompassing Exons 1-10 (Fig. 3B). Sequencing of the cDNA demonstrated the presence of the c.59del maternal allele but absence of the paternal (wildtype at position c.59) allele. Aberrant long isoform transcripts from the paternal allele carrying the c.859 + 4 A > G variant presumably were degraded through nonsense-mediated mRNA decay (NMD) (Fig. 3C left panel). This was confirmed with another RT-PCR fragment spanning exon 3–11 (data not shown). *LDB3* exon 9 is the last exon of the short isoform – ZASP-S, and only the c.59del variant is present in the short transcript. Sequencing of the cDNA showed bi-allelic expression at the c.59 position in the patient (Fig. 3C left panel). This indicates that mRNA from the c.859 + 4 A > G variant-carrying paternal allele is not degraded through NMD in the short isoform. Sequence analysis of the short-isoform-specific RT-PCR fragments showed aberrant transcripts where the first 110 intronic nucleotides are spliced in between the neighboring exons (Fig. 3C right panel). Splice prediction tools (from Alamut) show the presence of a cryptic splice donor at this position that likely replaces the inactivated canonical splice donor for intron 7.

In summary, no expression of the long *LDB3* isoform would be expected in the muscle tissue from the fetus; except perhaps a very short protein of 40 amino acids – if viable - from the c.59del allele. This is only allele that is detected in the mRNA, indicating that long transcripts from the allele with the c.859 + 4 A > G variant are degraded, and there will likely be no LDB3 proteins with the functional C-terminal LIM domain. There may however be expression of the short albeit mutated isoform, in which the PDZ domain is predicted to be present (Fig. 3D, E).

### Family 2

The proband, the youngest of four children born to a first-cousin couple of Iranian origin presented with elevated AST (aspartate aminotransferase) levels after birth and ultrasound scan showed hepatomegaly. Echocardiogram at 1 month old showed mild TR/MR (tricuspid/mitral regurgitation), PFO (Patent Foramen Ovale), dilated left ventricle and left atrium with a poor LVEF (Left Ventricular Ejection Fraction) (10–15%). She was diagnosed with dilated cardiomyopathy with LV non compaction. Her older brother died due to cardiac arrest with similar presentation at 40 days. WES of the proband revealed an ultra-rare (allele frequency: 0.000004013) homozygous nonsense variant in *LDB3*, NM_007078.2:c.1621C > T; NP_009009.1:p.(Arg541Ter), residing in a 5.62 Mb region of homozygosity (ROH). Both parents and one unaffected sister who were tested for the variant by Sanger sequencing were all carriers (Fig. 1B). This variant is present only in the long LDB3 isoform (NM_007078.2). WES did not identify any additional variants of interest in Family 2. According to ACMG guidelines the variant was assigned as a Variant of Uncertain Significance (c.1621C > T p.(Arg541*): PM2; PM3_P; PP1_P; PP3: VUS).

### Family 3

The proband is an infant daughter of a Saudi Arabian first-cousin couple who presented in the neonatal period with shortness of breath and feeding difficulty and was diagnosed to have dilated cardiomyopathy at the of age of 1 month. She has a healthy brother and there is no family history of inherited cardiac disease in the family. She had recurrent admissions with chest infections and was found to have ejection systolic murmur along the left sternal border. Electrocardiogram showed sinus tachycardia and there was moderate to severe cardiomegaly with mild congestion of the lung visible on chest X-ray. Echocardiogram revealed severely dilated left ventricle and left atrium with mild mitral valve regurgitation. Ejection fraction was less than 20%. Cardiac catheterization excluded coronary abnormalities. Plasma acylcarnitine profile, creatine kinase, and serum lactate levels were normal. She was treated with multiple anti-heart failure medications. However, she continued to have recurrent admissions with chest infection and heart failure symptoms. She succumbed to her disease at the age of 19 months. WES identified the same homozygous *LDB3* nonsense variant as found in family 2, NM_007078.2:c.1621C > T; NP_009009.1:p.(Arg541Ter). Her parents were heterozygous, and her unaffected brother was homozygous for the wild-type variant (Fig. 1C). WES did not identify any additional variants of interest in Family 3.

### Family 4

The proband is an 8-year-old Iranian girl born to reportedly unrelated parents who are from a small village. She presented with low LVEF and was diagnosed with cardiomegaly at the age of 5 years old. Her siblings died at the age of 25 and 31 months old with cardiomegaly and cardiomyopathy. WES of the proband identified a novel homozygous frameshift variant in *LDB3*, NM_007078.2:c.1881_1884del; NP_009009.1:p.(Gln627HisfsTer75) residing within an 8 Mb ROH. There were no available DNA samples from the deceased siblings for segregation analysis (Fig. 1D). WES did not identify any additional variants of interest in Family 4. According to ACMG guidelines the variant was assigned as a Variant of Uncertain Significance (c.1881_1884del p.Gln627Hisfs*75: PM2; PM3_P; PP3: VUS).

### Family 5

The proband is a 5-week-old male boy of Mexican ancestry born to parents who are third cousins (Fig. 1E). Pregnancy was complicated by pre-eclampsia and delivery was via c-section at 36 weeks of gestational age. The proband presented with sudden onset poor oral intake, respiratory difficulty, and lethargy. In the emergency room exam was notable for mottled appearance, sunken fontanelle, subcostal retractions, and lack of response. The initial blood gas showed a pH 6.8, CO_2_ 35, bicarbonate of 6 and lactate 14.9. Other laboratory abnormalities included transaminitis and coagulopathy (INR 4.7). Patient was intubated and chest x-ray showed cardiomegaly. Echocardiogram showed moderate left ventricular dilation with severely depressed left ventricular function (EF 14%) and moderate right ventricle depressed function (Fig. 2E–H). Patient was started on epinephrine and milrinone drips and furosemide for diuresis with improvement of perfusion and lactate to 1.4. Newborn screening, plasma acylcarnitine profile, and serum amino acids were normal. Genome study revealed a novel homozygous variant in *LDB3* NM_007078.2:c.1219_1222del; NP_009009.1:p.(Val407ThrfsTer84). Parents were both heterozygous for the *LDB3* variant with no history of cardiac disease. WES did not identify any additional variants of interest in Family 1. According to ACMG guidelines the variant was assigned as a Variant of Uncertain Significance (c.1219_1222del p.(Val407Thrfs*84): PM2; PM3_P; PP3: VUS). The proband is in the cardiac intensive care unit tolerating transition to oral heart failure medications (Captopril, Aldactone, Digoxin, Propranolol) and monitoring weight gain.

## Discussion

In this report, we provide molecular and clinical data demonstrating that recessive loss-of-function *LDB3* variants lead to a severe early-onset cardiomyopathy with additional skeletal muscle involvement likely dependent on the location of the variant.

Multiple Cypher/ZASP isoforms encoded by *LDB3* exist and are believed to play unique roles in maintaining the Z-disc structure in heart as well as skeletal muscle. Both global and cardiac specific Cypher-null mice display premature lethality associated with severe dilated cardiomyopathy and heart failure, characterized by severely disorganized and disrupted Z-lines in striated muscle [12, 13, 17]. Furthermore, morpholino knockdown of Cypher in zebrafish also results in a severe dilated cardiomyopathy phenotype [18].

In humans, autosomal dominant missense variants in *LDB3* that affect only short isoforms are associated with skeletal myopathies [9], while variants affecting only long isoforms are primarily associated with cardiomyopathies [8, 19]; however bi-allelic or loss-of-function *LDB3* variants have yet to be reported [3].

The loss-of-function variants found in Family 1 are present in both long and short isoforms of LDB3 and were associated with highly irregular Z-disc formation seen in skeletal muscle tissue from the fetus, in addition to cardiomegaly. In contrast the variants discovered in families 2-5 are only present in the long isoform, and there were no indications of muscular dystrophy in these families, highlighting the importance of the PDZ domain in skeletal-muscle specific isoforms.

Recessive inheritance in our five families was also associated with a more severe phenotype than that reported for dominant variants. This is in line with other reports of recessive variants in classically autosomal dominant cardiomyopathy genes [20, 21]. Interestingly heterozygous carriers of the variants were asymptomatic—although it should be noted that the parents of Family 1 declined cardiac examination, suggesting that heterozygous missense variants in the *LDB3* gene may be more deleterious than heterozygous loss-of-function variants in this gene. Indeed, the Probability of Loss-of-function Intolerance (PLi) score of the *LDB3* gene is <0.9, indicating that this gene can tolerate protein truncating variations (https://gnomad.broadinstitute.org) [22].

In conclusion, we find that recessive loss-of-function *LDB3* variants can lead to an early-onset and lethal human phenotype of cardiomyopathy and myopathy. Further studies will be required to fully elucidate the underlying molecular mechanisms and potentially tissue-specific genotype-phenotype correlations.

## Data Availability

The datasets generated and/or analysed during the current study are available from the corresponding author on reasonable request.
